# The relationship between the alterations in metabolite levels in the dorsolateral prefrontal cortex and clinical symptoms of patients with first-episode schizophrenia: a one year follow-up study

**DOI:** 10.18632/oncotarget.23983

**Published:** 2018-01-04

**Authors:** Manli Huang, Wuqiu Guo, Shaojia Lu, Fen Pan, Jinkai Chen, Jianbo Hu, Shaohua Hu, Weijuan Xu, Desheng Shang, Yi Xu

**Affiliations:** ^1^ Department of Psychiatry, First Affiliated Hospital, College of Medicine, Zhejiang University, The Key Laboratory of Mental Disorder’s Management of Zhejiang Province, Hangzhou 310003, China; ^2^ Department of Psychology and Behavioral Science, Zhejiang University, Hangzhou 310028, China; ^3^ Department of Radiology, First Affiliated Hospital, College of Medicine, Zhejiang University, The Key Laboratory of Mental Disorder’s Management of Zhejiang Province, Hangzhou 310003, China

**Keywords:** dorsolateral prefrontal cortex (DLPFC), MRS, PANSS, schizophrenia, N-acetylaspartate (NAA)

## Abstract

**Background:**

Reduced brain metabolites such as N-acetyl-aspartate (NAA), glutamate (Glx), Choline (Cho) and myo-inositol (MI) have been repeatedly found in first-episode schizophrenia (FES) and suggest neuronal loss or dysfunction. However, the potential relationship between the metabolite level and the clinical symptoms or the recovery of FES remained unclear.

**Objectives:**

This study aimed to investigate the correlation between the alterations in dorsolateral prefrontal cortex (DLPFC) metabolite levels of patients with first-episode schizophrenia (FES) and the changes in clinical symptoms after one year treatment.

**Materials and Methods:**

FES patients underwent 1H-MRS scan twice: one time at the baseline and the other one year later, while the healthy group patients underwent only once at the baseline time. The symptom severity of patients was measured by PANSS.

**Principal Observations:**

An increase in the NAA/Cr level was detected in the left DLPFC of patients with FES. The change in the NAA/Cr level was significantly correlated with the alteration in their PANSS-P score. The Cho/Cr levels on both sides of DLPFC in patients with FES were lower compared with the healthy controls both at the baseline and after the treatment. The NAA/Cr and MI/Cr levels in the right DLPFC were decreased after the treatment.

**Conclusions:**

(1) the depletion of NAA in left DLPFC might be a state characteristic; (2) the Cho/Cr level might be the potential endophenotype of schizophrenia; (3) the decrease of NAA/Cr and MI/Cr level in right DLPFC might be due to the development of schizophrenia.

## INTRODUCTION

Schizophrenia (SC) is a mental disorder characterized by abnormal social behavior and failure to understand reality. Common symptoms include false beliefs, unclear or confused thinking, hearing voices that others do not hear, reduced social engagement and emotional expression, and a lack of motivation [1]. The Positive and Negative Syndrome Scale (PANSS), a widely used clinical assessment for severity of SC symptoms, classified the typical symptoms of SC into three dimensions: positive, negative, and general syndromes [2].

With the rapid development of brain image techniques, the relationship between brain metabolites and clinical manifestation of schizophrenia has become a popular perspective to explore the mechanism of SC. Magnetic resonance spectroscopy (MRS), a technique that exploits the magnetic properties of certain atomic nuclei [3], can reflect the concentration of brain metabolites, and thus reveal the activation and number of neuron cells in brain cortex [4].

The metabolites selected by the researchers have two important characteristics in common: they mark the number or the function of neuron cells [5, 6] and they are easily detected by the MRS device [7]. For instance, N-acetylaspartate (NAA), synthesized in neuronal mitochondria from acetyl-coenzyme A and aspartate by the enzyme NAA transferase, has been considered to be a marker of neuronal integrity and an indicator for the number of viable neurons [8–10]. Also, glutamate is the agonist of N-methyl-D-aspartate (NMDA) receptors, which play an important role in excitatory neurotransmission, plasticity, and excitotoxicity. The activation of NMDA receptors is considered to be correlated with the positive and negative syndromes of SC [11–15]. Choline (Cho), generated from the cell membrane phospholipids, which reflects the density of neuron cells, and myo-inositol (MI), considered to be the agonist for Ca2+release in mitochondria and endoplasmic reticulum, which reveals the function of neuron cells and astrocytes, both interest the scientists [16–19].

Dorsolateral prefrontal cortex (DLPFC) was considered to be associated with cognitive function [20, 21]. It has been indicated that the regional cerebral blood flow in DLPFC may help to distinguish patients with SC and normal subjects when they are involved in the same cognition task (no matter what the task is). Also, the better the DLPFC is able to function, the better the cognition performance recorded by the patients with SC [22–24]. Based on the physiological and neuroimaging findings on DLPFC in patients with SC, it is meaningful to detect the neuron-metabolite level in DLPFC to understand the mechanism of SC.

The number of researches using MRS to explore the pathophysiology of SC has grown rapidly in recent years. Most studies have reported a decreased metabolite level in DLPFC among patients with SC, comparing to the healthy controls [25]. Depletion of metabolite of NAA [26], Glx [27], Cho [16] or MI [19] in DLPFC of patients with SC has been widely studied by researchers, which are thought to reflect the dysfunction of schizophrenia-related neuron cells [28]. However, few studies have explored whether the change in certain metabolite is correlated with the process of disease or its medical treatment. As a matter of fact, it is necessary to perform long-term longitudinal studies to explore the problem.

Longitudinal studies carried out before majorly focused on the alteration of metabolite level after a period of time or were for the purpose of pharmacology [29, 30]. However, few studies had discussed the correlation between the metabolite level and the severity of clinical symptoms of SC. The results given out by previous studies about the correlation between the metabolite level and the PANSS score were unstable: Tanaka et al [31] reported that there was significant negative correlation between the NAA level in DLPFC and PANSS-N scores while Sigmundsson et al. [32] reported a positive correlation between the NAA level in right frontal lobe and PANSS-P scores, however, other researchers did not observe significant correlation [33, 34]. Meanwhile, most previous longitudinal studies were carried out within no longer than 3 months [6, 8, 33, 35], the change of metabolite level in a longer treatment period remained unexplored. Moreover, few studies were carried out to explore the change of metabolite level after the treatment of SC in mainland China.

The aim of the study was to investigate the correlation between the alterations in dorsolateral prefrontal cortex (DLPFC) metabolite levels of Chinese patients with first-episode schizophrenia (FES) and the change in clinical symptoms after one year treatment to explore the pathophysiology of SC.

## MATERIALS AND METHODS

### Subjects

**Figure 1:**
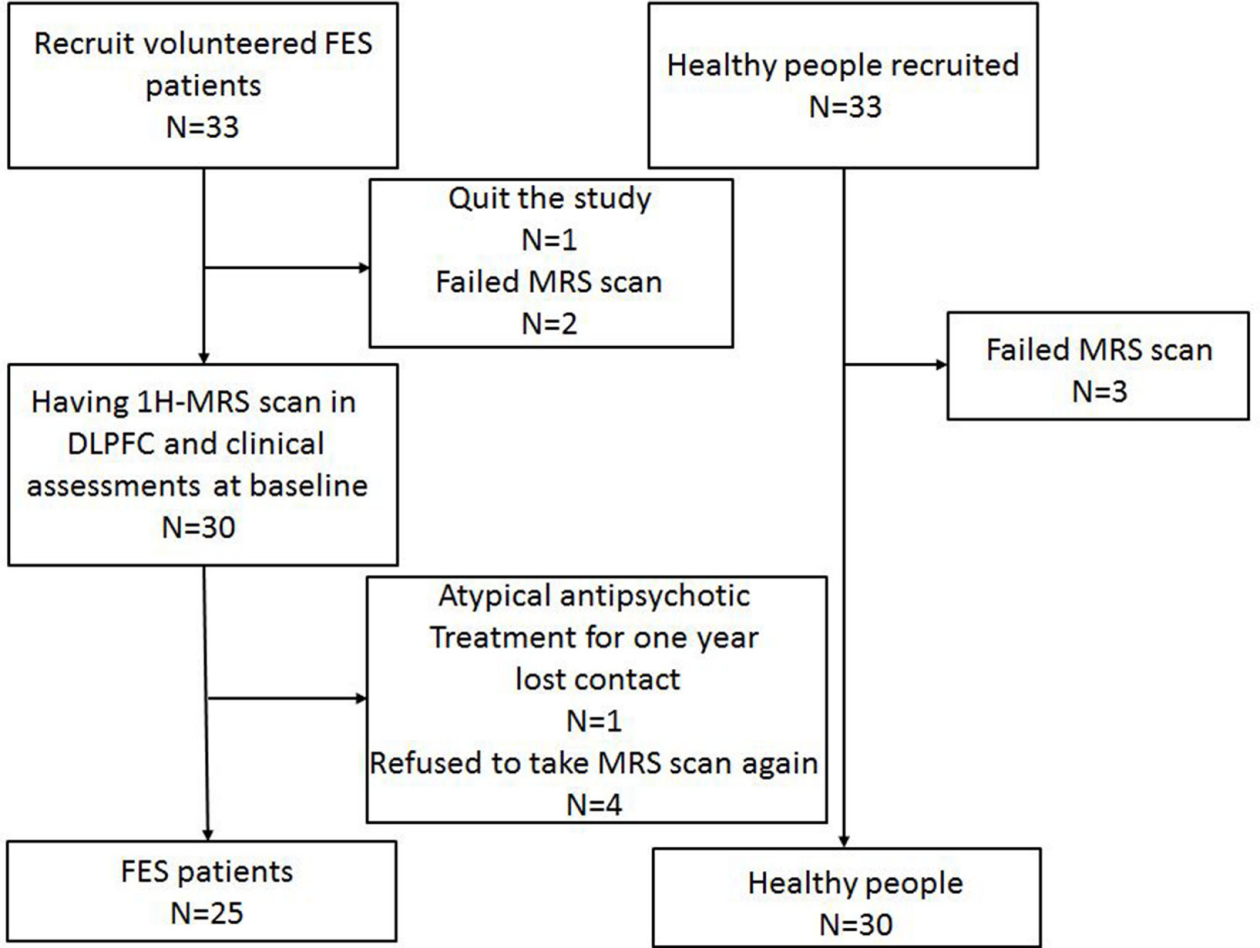
CONSORT flow chart

A total of 33 patients were recruited from the First Affiliated Hospital of Medical School of Zhejiang University, while 33 healthy people were recruited from communities or through advertisements. Routine laboratory tests and physical and neurological examinations were administered to each participant. One patient decided to quit the study right before the MRS scanned, two patients as well as three healthy people failed to finish the MRS scan at the baseline. One patient lost contact during the one-year follow up and four patients refused to have a MRS scan again. Therefore, the statistics of 25 patients with FES and 30 healthy people were included in the final statistical analysis (Figure 1).

The inclusion criteria were as follows: (1) met the diagnostic criteria for schizophrenia in the International Statistical Classification of Diseases and Related Health Problems, 10th revision [International Classification of Diseases (ICD)-10 F20; ICD-10 diagnosis in all studies verified by the mini-International Neuropsychiatric Interview (MINI)] (World Health Organization, 1992); (2) first episode; (3)being antipsychotic drug-naïve; (4) ethnicity of Han origin; and (5) right-handed. The exclusion criteria for both patients and healthy people were as follows: (1)with a primary active ICD-10 diagnosis other than schizophrenia at screening or an ICD-10 diagnosis of active substance dependence within 3 months before screening (except nicotine and caffeine); (2) with a diagnosis of past psychiatric or central nervous system disorders; (3) any contraindications to MRS scanning(e.g., claustrophobia or metallic implants); (4) with a diagnosis of serious disease of the heart, liver, kidney, internal secretion, blood system or any other disease that might disturb the outcome of the study; (5) with a diagnosis of organic mental disorders or mental retardation; and (6) pregnancy.

The study was approved by the ethics committee of the First Affiliated Hospital of Medical School of Zhejiang University. All subjects provided written informed consent before participating in the study. The trial was conducted in accordance with the ethical principles included in the Declaration of Helsinki consistent with Good Clinical Practices and applicable regulatory requirements. All subjects were informed that they could quit the study at any time with an additional examination and further therapy support. The clinical trial number of the study is ChiCTR-COC-14005302.

### Study design

This was a one-year longitudinal research to investigate the correlation between the alterations in dorsolateral prefrontal cortex (DLPFC) metabolite level of patients with drug-naïve first-episode schizophrenia (FES) and the change in clinical symptoms.

The first patient was included to the study in January 2014, and the last patient was included in November 2014. The healthy controls were recruited during the period. The patients were followed for at least one year when they were taking atypical antipsychotic therapies. The study was totally finished in December 2015.

All patients fulfilled their demographic data, and both groups underwent an 1H-MRS scan in DLPFC at the baseline. The initial clinical characteristics of the FES patients were recorded with PANSS. All patients received antipsychotic drugs for one year, including risperidone, paliperidone, quetiapine, and olanzapine, and were reevaluated at week 1, 2, 3, 4, 8, 12, 24, 36, and 48 with PANSS during the year. After 1-year treatment, 1H-MRS of the patients was reevaluated and recorded.

### Clinical assessments and MRS acquisition

#### Clinical assessments

PANSS was used to evaluate the clinical characteristics of the subjects in the schizophrenia group. The PANSS is a 30-item medical scale including 7 positive symptoms (delusions, conceptual disorganization, hallucinations, hyperactivity, grandiosity, suspiciousness/persecution, and hostility), 7 negative symptoms (blunted affect, emotional withdrawal, poor rapport, passive/apathetic social withdrawal, difficulty in abstract thinking, lack of spontaneity and flow of conversation, and stereotyped thinking), and 16 general psychopathology items (somatic concern, anxiety, guilt feelings, tension, mannerisms and posturing, depression, motor retardation, uncooperativeness, unusual thought content, disorientation, poor attention, lack of judgment and insight, disturbance of volition, poor impulse control, preoccupation, and active social avoidance). It is used to assess the degree of psychopathology on a number of symptomatic domains. Each item is rated from 1 (no evidence) to 7 (extreme) based on objective criteria. The reliability of PANSS scale in chinese version is 0.8707 and the vadility is above 0.7434 [36].

### MRS acquisition

**Figure 2:**
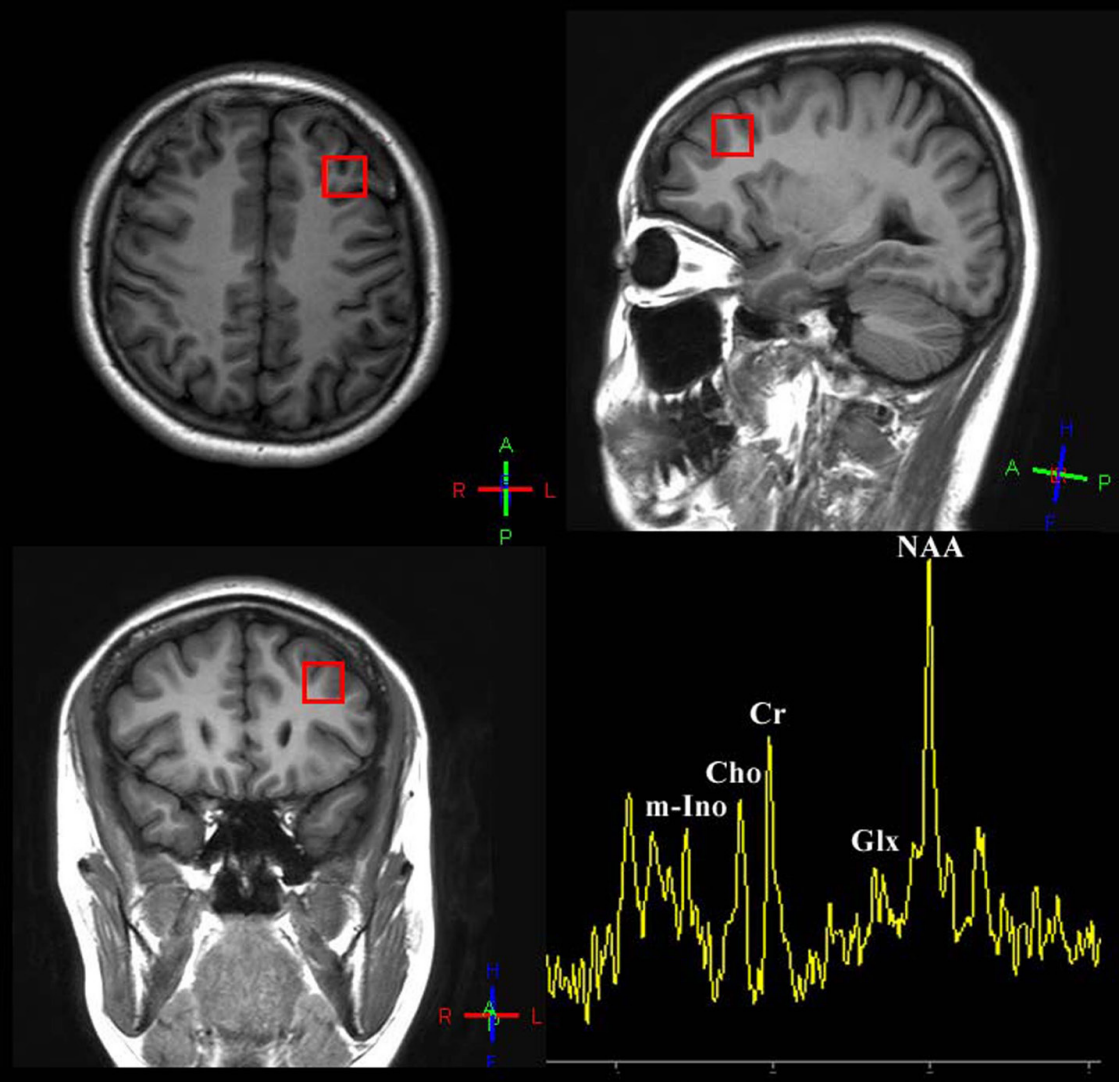
Voxel placement of interest: dorsolateral prefrontal cortex and a sample of quantifying 1H-spectrum acquired from DLPFC of patients/healthy people

All MRI and MRS examinations were performed on a 3.0-Tesla MR scanner (Achieva 3.0T; Philips Medical Systems, Netherlands). Routine maintenance was performed to the scanner every few month, and patients were scanned on the same scanner after a year. Water model experiments were performed every two months, the signal-noise ratios of water model were all above 200, which assured the reliability of the scanner. The system was tested for data stability prior to use. A standard coil was used to emit and receive magnetic resonance imaging signals. Head movement was reduced using foam pads, and earplugs were used to reduce noise stimulation. The subjects were instructed to relax, keep their eyes closed, stay awake, remain still, and not think of anything in particular. The subjects’ compliance was confirmed after the scanning was completed. Spectroscopy data were acquired from a single voxel using chemical shift selective saturation (for water suppression) stimulated echo pulse with the following acquisition parameters: echo time = 9.2 ms; repetition time = 2000 ms; mixing time = 16ms; volume of interest(VOI) = 15 × 15 × 15 mm3; number of signal average = 128; and sample = 1024. The volume of interest (VOI) was prescribed to include mostly grey matter in the left and right dorsallateral prefrontal cortex using coronal, sagittal and transverse image as shown in Figure 1. A three-plane localizer MRI was first acquired to define the spatial position of the brain. Three-plane oblique localizer MRI was then acquired: an axial/oblique MRI series parallel to the Sylvian fissure, a coronal/oblique localizer MRI series perpendicular to the previous axial/oblique planes, and sagittal/oblique MRI series. To ensure correct slice prescription, the anterior commissure was located on a coronal/oblique image, and the sagittal/oblique slices were oriented parallel to the brain surface at the middle frontal gyrus, forming a 45° angle (approximately) with the interhemispheric fissure (Figure 2).

The standard spectroscopic phantom was used to determine reliability before spectrum scanning. The line width was less than 4 Hz and water suppression level was at least 99% in the pre-scan for patients. The data post-processing (including signal-to-noise ratio assessment and baseline adjustment) and quantification steps were automated performed by the Spectrum View. The quantitative data of NAA and Glx (major metabolites), choline-containing compounds, and MI (minor metabolites) were calculated using the Spectrum View.

### Statistical analysis

All analyses were performed using the statistical package SPSS version 17.0 (SPSS, IL, USA). Differences in demographical characteristics and the index of 1H-MRS at the baseline between control and patient groups were determined using the independent *t*-test or χ^2^ test. The paired *t*-test (two-tailed) was used to evaluate significant changes in the index of 1H-MRS, neuropsychological assessment results, and clinical symptoms before and after an atypical antipsychotic treatment. Partial correlation analysis was used to calculate the correlations between the metabolite alteration and pathological characteristics or cognition condition (set baseline metabolite concentration as the unconcerned confounding factor). The level of significance was set at α = 0.05.

## RESULTS

### Demographic data and clinical symptoms

As shown in Table 1, no significant difference was found in all the domains of demographic statistics including age, gender, nationality, and education years between patients with schizophrenia and the healthy subjects.

**Table 1:** Demographic and clinical characteristics of schizophrenia group and control group at the baseline and 1 year later

Measure	Schizophrenia (*N* = 25)		Control (*N* = 30)	*t*/χ^2^	*p*
	Baseline	1 year			
Age, mean ± SD (year)	19.92 ± 4.65		19.23 ± 4.41	0.568^a^	0.573
Gender (male/female)	12/13		16/14	0.155^b^	0.694
Nationality, Han/other (*n*)	25/0		30/0		
Education years	11.40 ± 3.08		11.65 ± 3.51	–1.422 ^a^	0.164
Course of illness (mo)	12.28 ± 13.54				
Age at first onset (year)	18.12 ± 4.47				
PANSS total scores	80.83 ± 15.64	50.50 ± 18.39		6.154	< 0.001^***^
PANSS-P scores	19.87 ± 7.45	12.33 ± 5.19		4.067^a^	< 0.001^***^
PANSS-N scores	37.25 ± 11.34	14.79 ± 6.76		4.310^a^	< 0.001^***^
PANSS-G scores	24.67 ± 9.21	23.54 ± 10.87		0.387^a^	0.701

^a^*t* test; ^b^χ^2^ test;^*^P <0.05; ^**^P <0.01; ^***^P <0.001.

**Figure 3:**
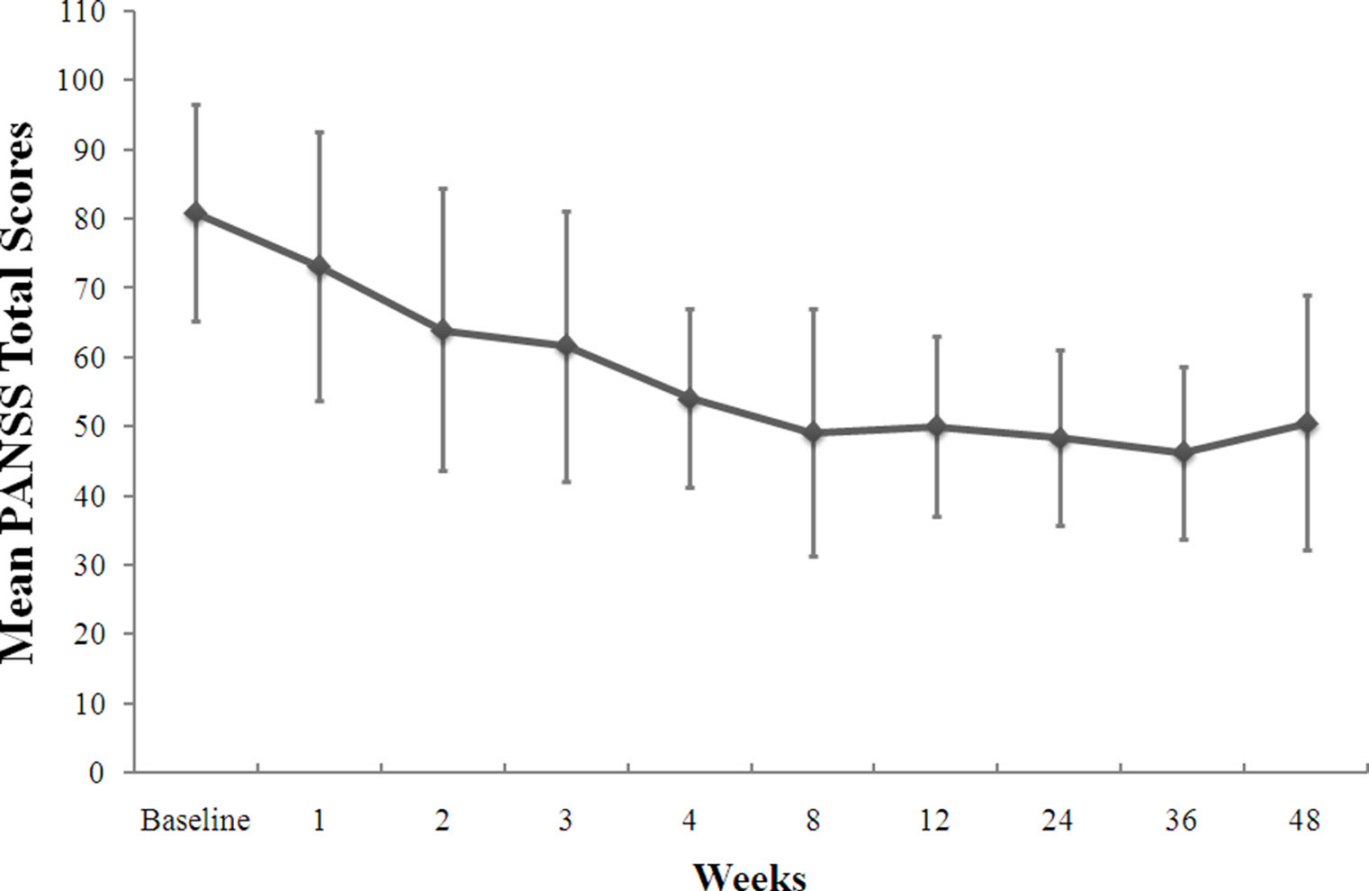
Change from the baseline in PANSS total scores during the 1-year treatment

The clinical symptoms, which were measured using PANSS, were significantly improved after 1-year treatment. The line chart of the PANSS score recorded at every time point is shown in Figure 3. The PANSS total score and the subscales of PANSS-P score and PANSS-N score all showed a significant reduction after one year treatment However, there was no significant difference in the PANSS-G score after the treatment compared with the baseline (Table 1).

### MRS data

Table 2 showed that at the baseline, a lower ratio of NAA/Cr was observed in the left DLPFC of the FES group compared with the healthy group (t(24) = –2.222; *P* = 0.031). The same result was also obtained for the Cho/Cr level in the left (t(24) = –3.363, *P* = 0.001) and the right lobe (t(24) = –2.762, *P* = 0.008). Other metabolite levels showed no significant changes.

**Table 2:** ^1^H-MRS statistics of schizophrenia group and control group at the baseline and 1 year later

Measure	Baseline	1 year	Control	t^a^	*P*	t^b^	*P*	t^c^	*P*
Left NAA/Cr	1.89 ± 0.42	2.15 ± 0.69	2.34 ± 0.93	–2.222	0.031^*^	-0.887	0.379	–1.572	0.122
Left Ml/Cr	0.92 ± 1.18	0.87 ± 0.39	0.75 ± 0.50	0.409	0.520	0.966	0.339	0.199	0.844
Left Glx/Cr	1.84 ± 1.15	1.72 ± 0.93	1.43 ± 0.56	1.630	0.113	1.370	0.179	0.405	0.688
Left Cho/cr	0.83 ± 0.26	0.88 ± 0.27	1.19 ± 0.48	–3.363	0.001^*^	–2.836	0.006^*^	-0.712	0.480
Right NAA/Cr	1.91 ± 0.72	1.62 ± 0.45	2.16 ± 0.58	–1.351	0.183	–3.817	< 0.001	1.677	0.101
Right Ml/Cr	2.05 ± 2.14	0.83 ± 0.46	1.88 ± 2.11	0.297	0.768	–2.425	0.019^*^	2.766	0.008^*^
Right Glx/Cr	1.55 ± 0.74	2.24 ± 2.13	1.38 ± 0.87	0.787	0.435	1.897	0.067	–1.532	0.136
Right Cho/cr	0.72 ± 0.24	0.72 ± 0.28	1.12 ± 0.67	–2.762	0.008^*^	–2.731	0.009^*^	0.032	0.975

*P < 0.05.

^a^Comparison of baseline and control;

^b^comparison of one year later and control.

^c^Comparison of baseline and one year later;

Both *t*^a^ and *t*^b^ used independent-sample *t* test, *t*^c^ used paired *t* test.

After a one-year treatment, the NAA/Cr level in the left DLPFC showed no significant difference compared with the control group (t(24) = 0.887, *P* = 0.379). However, the Cho/Cr on both sides did not change significantly compared with the control group after the treatment (left: t(24) = 2.836, *P* = 0.006; right: t(24) = 2.731, *P* = 0.009). Meanwhile, the NAA/Cr and MI/Cr levels in the right lobe were significantly lower compared with the control groups (NAA: t(24)=3.871, *P* < 0.001; MI: t(24) = 2.425, *P* = 0.019).

Compared with the baseline, the MI/Cr level in the right DLPFC of patients with FES showed a significant decrease (t(24) = 2.766, *P* = 0.008), while the levels of other metabolites showed no significant difference.

### Correlation analysis

**Figure 4:**
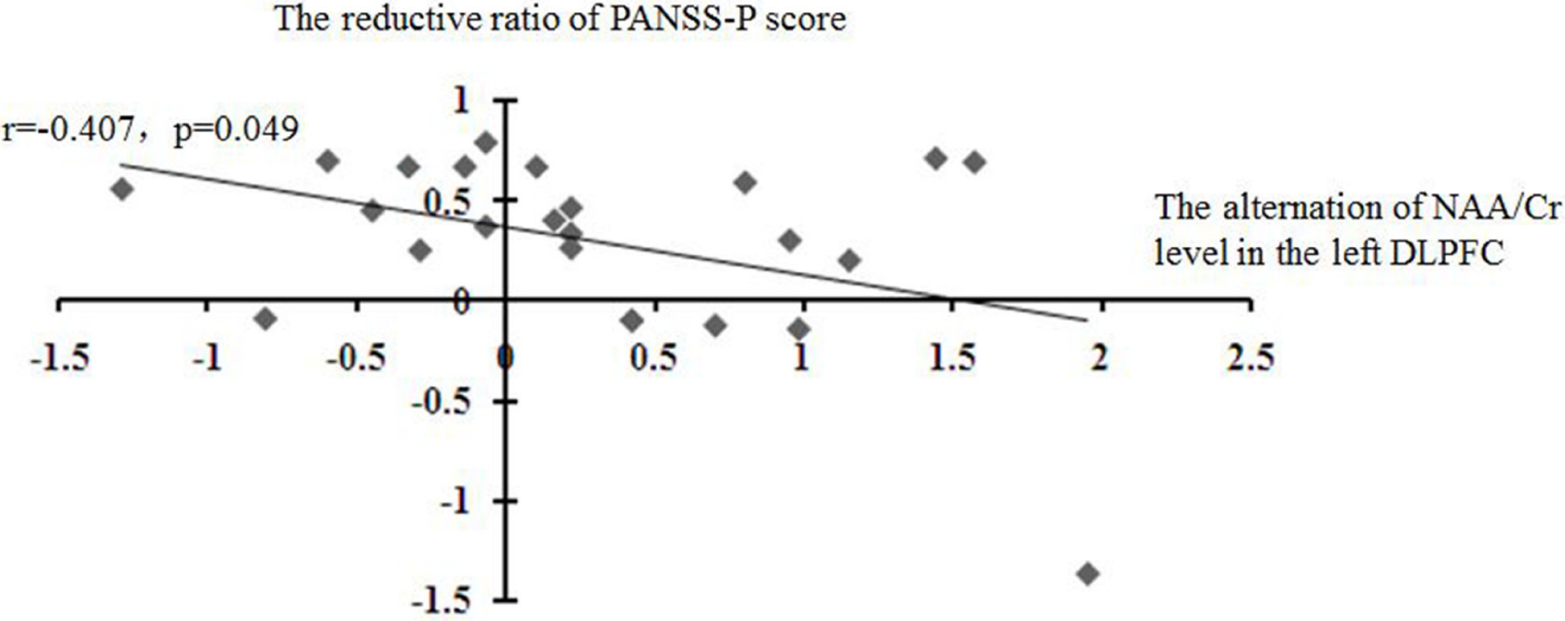
Correlations of the alteration of the NAA level in the left DLPFC with the reduction ratio of PANSS-P score

After the treatment, a significant negative correlation was observed between the alterations of the NAA/Cr levels in the left DLPFC and the PANSS-P reduction ratios (*r* = -0.407, *P* < 0.05) (Figure 4).

Nonetheless, this study did not find any correlations between the change in the levels of other metabolites and PANSS score reduction or any other demographic statistics (Table 3).

**Table 3:** Correlation analysis between the alternation of MRS statics and the reductive ratio of PANSS scores

Measure	Left NAA/Cr	Left Ml/Cr	Left Glx/Cr	Left Cho/Cr	Right NAA/Cr	Right Ml/Cr	Right Glx/Cr	Right Cho/Cr
PANS-T reductive ratio	−0.285	0.089	0.072	0.097	−0.222	−0.029	−0.177	−0.040
*p*	0.176	0.678	0.738	0.651	0.298	0.894	0.408	0.852
PANSS-P reductive ratio	−0.407	−0.002	−0.151	0.093	−0.177	−0.145	0.090	0.027
*p*	0.049^*^	0.992	0.481	0.665	0.409	0.498	0.677	0.899
PANSS-N reductive ratio	0.320	−0.068	0.118	−0.006	0.000	−0.146	−0.142	0.073
*p*	0.127	0.751	0.581	0.978	1.000	0.496	0.507	0.735
PANSS-G reductive ratio	−0.223	0.184	0.290	0.068	−0.239	0.328	−0.233	−0.066
*p*	0.294	0.390	0.169	0.751	0.260	0.117	0.274	0.760

^*^P < 0.05.

## DISCUSSION

In this study, the clinical symptoms of FES patients were significantly improved after a one-year treatment. Compared with the baseline, the NAA/Cr level of FES patients in the left DLPFC showed a tendency to rise while the NAA/Cr and MI/Cr level in the right DLPFC showed a tendency to fall. The Cho/Cr level of FES patients was lower than the healthy control, both at baseline and one year later.

At the baseline, the NAA/Cr levels in the left DLPFC of patients with drug-naïve FES was significantly lower compared with the control groups, which was consistent with many other researches and the meta-analysis conducted by Marsman et al and Brugger et al [25, 26, 28, 37]. The difference in the NAA/Cr levels disappeared after the treatment, implying that the NAA/Cr level might change synchronously with the healing process. Several previous studies have shown that the NAA/Cr level in the left DLPFC increased significantly or had a tendency to increase after the atypical antipsychotic medical treatment [29, 30, 38]. In agreement with these results, the present study also showed an increasing trend of the NAA/Cr level in the left DLPFC after one-year treatment. However, the patients were followed up for 1 year in the present study, which was much longer compared with any of the previous studies with similar results. It is believed that the reproducibility of NAA-related neuron cells could be activated with the recovery of schizophrenia using a chronic atypical antipsychotic therapy [39], no matter the treatment lasted for 4 weeks [29], 8 weeks [30], or a year, suggesting that the depletion of NAA might be a state characteristic. More importantly, a significant negative correlation was observed between the alterations of the NAA/Cr levels in the left DLPFC and the PANSS-P reduction ratios, indicating that the rise in NAA levels might be associated with the change in the positive symptoms. On the contrary, a lower NAA/Cr level was found in the right DLPFC compared with the healthy people, while no significant difference was noted at the baseline, which differed from the increasing tendency in the left lobe after the treatment. Although the concrete function of the two lobes has not been explored, it was demonstrated that the left and right lobes of DLPFC played different roles in cognitive or emotional process for both healthy people and patients with SC [40–42]. Moreover, in most previous studies, only the NAA/Cr level in the left lobe, rather than both, of the DLPFC increased significantly or showed a tendency to rise after the treatment [29, 38, 43].

Compared with the healthy people, the patients with FES had significantly lower Cho/Cr levels in both lobes, and the levels remained unchanged after the treatment. Some other researches also yielded similar results [8, 35]. This might be reason that the depletion of Cho reflected a cortical impairment caused by schizophrenia and it did not recover easily despite the therapy, suggesting that Cho could be the potential endophenotype of SC.

MI was thought to be a marker of astroglial activity [18]. Also, the dysfunction or loss of astrocytes was considered to obstruct the glutamate metabolism system [44] and was widely found in patients with schizophrenia [19, 45, 46]. MI is the only metabolite whose level significantly changed after the treatment. Nonetheless, the reduction of the MI/Cr level in the right DLPFC was more a sign of worsening than a sign of recovery. Considering no significant differences between the two groups at the baseline were found, the huge reduction in the MI/Cr level in patients could not be explained by the recovery. Rather, it might be due to the development of SC.

This study did not find any significant difference in the Glx/Cr level between patients with FES and healthy people, whether at baseline or after the treatment. Although some studies reported the same finding. [47, 48], the outcome of this study was different from the meta-analysis performed by Marsman et al. [28]. According to Marsman [28], the Glx level in the frontal region was significantly lower in patients than healthy people. This discrepancy might be due to the small sample size, heterogeneity of schizophrenia, different measurement methods, enrollment criteria, or individual specialties.

This study had certain limitations. First, with the difficulty to recruit and follow up the Chinese patients with drug-naïve FES for 1 year in clinical researches; the sample size might be too small to get proper statistics, which might unavoidably affect some outcomes of this study. Second, the patients with FES recruited in this study took a different kind of medicine for the treatment. The sample size was too small to carry out an analysis of the effect of a different medicine on the metabolite level. Third, Most healthy people refused to have MRI after 1 year, so their MRS data were not reevaluated, which might somehow influence the result of this study. Finally, because of the lack of a standard research fitting package such as Linear combination of Model *in vitro* spectra (LC model), the concentrations of metabolites could not be calculated. As a consequence, only the ratios of metabolites to Cr were chosen as the dependent variables. Although the results using a dependent variable such as concentration or ratio are reported to be the same in most studies and meta-analyses, using only the ratio for analysis might challenge the reliability of outcomes of the present study.
